# Decision-making in structure solution using Bayesian estimates of map quality: the *PHENIX AutoSol* wizard

**DOI:** 10.1107/S0907444909012098

**Published:** 2009-05-15

**Authors:** Thomas C. Terwilliger, Paul D. Adams, Randy J. Read, Airlie J. McCoy, Nigel W. Moriarty, Ralf W. Grosse-Kunstleve, Pavel V. Afonine, Peter H. Zwart, Li-Wei Hung

**Affiliations:** a Los Alamos National Laboratory, Los Alamos, NM 87545, USA; b Lawrence Berkeley National Laboratory, One Cyclotron Road, Building 64R0121, Berkeley, CA 94720, USA; c Department of Haematology, University of Cambridge, Cambridge CB2 0XY, England

**Keywords:** structure solution, scoring, Protein Data Bank, phasing, decision-making, *PHENIX*, experimental electron-density maps

## Abstract

Estimates of the quality of experimental maps are important in many stages of structure determination of macromolecules. Map quality is defined here as the correlation between a map and the corresponding map obtained using phases from the final refined model. Here, ten different measures of experimental map quality were examined using a set of 1359 maps calculated by re-analysis of 246 solved MAD, SAD and MIR data sets. A simple Bayesian approach to estimation of map quality from one or more measures is presented. It was found that a Bayesian estimator based on the skewness of the density values in an electron-density map is the most accurate of the ten individual Bayesian estimators of map quality examined, with a correlation between estimated and actual map quality of 0.90. A combination of the skewness of electron density with the local correlation of r.m.s. density gives a further improvement in estimating map quality, with an overall correlation coefficient of 0.92. The *PHENIX AutoSol* wizard carries out automated structure solution based on any combination of SAD, MAD, SIR or MIR data sets. The wizard is based on tools from the *PHENIX* package and uses the Bayesian estimates of map quality described here to choose the highest quality solutions after experimental phasing.

## Abstract 2

Ten measures of experimental electron-density-map quality are examined and the skewness of electron density is found to be the best indicator of actual map quality. A Bayesian approach to estimating map quality is developed and used in the *PHENIX AutoSol* wizard to make decisions during automated structure solution.

## 1. Introduction

Structure solution in macromolecular crystallography is a multi-step procedure in which more than one plausible possibility often exists at the conclusion of each step. At the start of the process, one or more MAD, SAD, SIR or MIR data sets are collected and reduced to a list of indices and structure-factor amplitudes (Leslie, 1992 ▶; Kabsch, 1993 ▶; Otwinowski & Minor, 1997 ▶; Pflugrath, 1999 ▶). Even at this stage there are often several possibilities for the space group that must be considered. For each possible space group, the process con­tinues with finding a substructure containing heavy atoms or anomalously scattering atoms (Grosse-Kunstleve & Adams, 2003 ▶; Schneider & Sheldrick, 2002 ▶; Terwilliger & Berendzen, 1999*a* ▶,*b* ▶; Weeks *et al.*, 2003 ▶). There is often more than one plausible substructure at this stage. For example, in space groups that are not chiral the two possible hands of the substructure cannot normally be distinguished. Furthermore, for MAD data sets there may be alternative solutions found by searching for the substructure using different data sets (from various wavelengths or combining data from different wavelengths using *F*_A_ values; Terwilliger, 1994 ▶). Similarly, for MIR data sets there may also be substructures found for several different derivatives. In addition to these intrinsic possibilities, it is possible that more than one set of parameters or even more than one set of software might be used to generate possible solutions. The potential heavy-atom substructures found are then used to calculate the phases of structure factors, which are in turn used as the starting point for density modification (Wang, 1985 ▶) and subsequent model building (*e.g.* Perrakis *et al.*, 1999 ▶; Terwilliger *et al.*, 2008 ▶). Normally, one of the best indications of map quality is that the map can be interpreted in terms of an atomic model.

If every possibility at every stage were investigated fully by calculating maps, carrying out density modification and model building, the process might take many hours or days to complete. To speed up the process, the possibilities at each stage are generally ranked, with only the highest ranked possibilities being considered for the next step. This approach can be efficient, but if it is to yield the best solution at the end it requires a reliable method for deciding which members of a set of solutions are of the highest quality.

The definition of ‘quality’ when applied to electron-density maps normally refers to the correlation between the values of electron density in the map and the values of electron density in a hypothetical ‘true’ map for the same structure. In this work, when tests are carried out to assess various measures of map quality, the ‘true’ quality or map correlation is calculated between the map in question and a map obtained using measured amplitudes but with phases calculated from a refined model of the corresponding structure. Maps that have a high map correlation as defined in this way are generally more useful for model building and interpretation than those with a low map correlation. However, it should be noted that map correlation is not a perfect way to assess the utility of a map, as low-resolution terms are generally stronger and therefore have a higher relative contribution to the correlation than high-resolution terms, while the high-resolution terms are generally essential for the interpretation of a map. Consequently, a map could have a moderately high correlation to a model map, based largely on low-resolution terms, yet not be interpretable.

A number of methods for evaluating the quality of experimental macromolecular electron-density maps have been developed. The methods can generally be grouped into real-space calculations and reciprocal-space calculations.

Real-space methods are based on an examination of the electron-density map and generally answer the question ‘Does this map look like an electron-density map of a macromolecule?’ There are many distinctive features of macromolecular electron-density maps that can be used to answer this question. A good map may be expected to have continuous chains of density (Baker *et al.*, 1993 ▶). It may have local patterns of density that reflect shapes and interatomic spacings common to macromolecules (Colovos *et al.*, 2000 ▶; Terwilliger, 2003 ▶). It may have a distribution of electron densities with a positive skewness, reflecting the large number of points with moderate or low electron density, the lack of points with negative density and the points with very positive electron density located near atoms in the structure (Podjarny, 1976 ▶; Lunin, 1993 ▶). There may be a large variation (contrast) in the local r.m.s.d. of electron density, reflecting regions of the structure containing the macromolecule (with high local variation) and solvent (with low local variation; Terwilliger & Berendzen, 1999*a* ▶; Sheldrick, 2002 ▶). The contiguous nature of the regions of relatively flat solvent may be detected from the correlation of local r.m.s.d. at one point in a map with that at neighboring points (Terwilliger & Berendzen, 1999*b* ▶). If noncrystallographic symmetry is present in the structure, then the correlation of NCS-related density can be detected (Cowtan & Main, 1998 ▶; Vellieux *et al.*, 1995 ▶; Terwilliger, 2002*a* ▶).

Reciprocal-space methods for evaluation of map quality generally address questions involving structure factors and expectations about the structure such as the model for the solvent region or for the heavy-atom substructure. One such question is simply ‘Given the anomalously scattering atom model and the observed data, what is the expected correlation between the experimental map and the true map?’ The value of the figure of merit of phasing (Blow & Crick, 1959 ▶; Terwilliger & Berendzen, 1999*a* ▶,*b* ▶), when estimated correctly, is similar in magnitude to the correlation between the experimental and true maps and can be used as an estimate of this correlation. Another question addresses the data and the expectations about the electron-density map: ‘Is the amplitude of each structure factor consistent with the value expected based on the amplitudes and phases of all other reflections and the model of the solvent region?’ This question can be answered based on the *R* factor in the first cycle of density modification (which reflects the agreement between each measured amplitude and an estimate of that amplitude based on all other amplitudes and phases along with expectations about features in the map; Cowtan & Main, 1996 ▶; Terwilliger, 2001 ▶). A related question can be asked about the phases: ‘If a phase is estimated from the model of the solvent region, measured amplitudes of structure factors and the experimental values of all other phases, is this phase correlated with its experimentally determined value?’ This question can be answered using the correlation of experimental phases with map probability phases obtained in statistical density modification (Terwilliger, 2001 ▶). A third question that might be asked is ‘Do the phases calculated using only the highest peaks in the map match the experimental phases?’ This question can be answered by truncating the density at a high level, calculating phases from the map and comparing these with the experimental phases (Baker *et al.*, 1993 ▶).

It is important to note that the measures of map quality are analyzed here for their utility in estimating the qualities of experimental electron-density maps, as opposed to maps that have been calculated using a partially correct model or maps that have had density modification applied. An important difference between experimental maps and those obtained using a model or based on density modification is that in the latter cases the maps have been specifically adjusted in order to maximize one or more of the properties that are being measured. For example, density modification typically flattens the solvent region of the map. Similarly, a map calculated from a model will tend to have a high skewness of the density values and a high connectivity of high electron density. Some of these measures may also be useful in these two other important cases, but the values of each measure corresponding to a particular quality of map are likely to be substantially different.

In this work, we implement ten different measures of quality of experimental electron-density maps, develop a simple Bayesian approach to estimating map quality from each and show how the individual estimates can be combined to yield useful overall estimates of map quality. These map-quality estimates are incorporated into the *PHENIX AutoSol* wizard and are used to make decisions during automated structure solution.

## 2. Materials and methods

### 2.1. Structure solution with the *PHENIX AutoSol* wizard

The *PHENIX AutoSol* wizard carries out structure solution for SAD/MAD or MIR/SIR/SIRAS data and any combination of these. If data representing more than one heavy-atom substructure were available, the data were grouped into ‘data sets’ with common heavy-atom substructures. All the structure solutions described here had been carried out previously and refined structures were available in each case. Default values were used here for most parameters, but the number and type of anomalous and heavy-atom scatterers and initial values of scattering factors were taken from this prior work.

#### 2.1.1. Analysis with *phenix.xtriage*

Each available set of data was analyzed using *phenix.xtriage* (Zwart *et al.*, 2005 ▶) for circumstances such as twinning, translational noncrystallographic symmetry, unexpectedly strong or weak reflections or groups of reflections or anisotropic overall atomic displacement parameters that may complicate structure determination. The data were corrected for anisotropy before structure solution was carried out if the overall anisotropy correction yielded values that were highly anisotropic (by default, defined as greater than a 1.5-fold ratio among the values of the parameters along the three principal reciprocal axes and greater than 20 Å^2^ difference between the highest and lowest values). If an anisotropy correction was applied, then the resulting corrected data were used for structure solution only and not for refinement (as an anisotropy correction is applied as part of the refinement process itself).

#### 2.1.2. Substructure solution with *HySS*

For each data set (*i.e.* a MAD or SAD data set or an SIR data set) possible heavy-atom substructures were found using the hybrid substructure search (*HySS*; Grosse-Kunstleve & Adams, 2003 ▶) from iso­morphous, anomalous or dispersive differences or from *F*_A_ values (Terwilliger, 1994 ▶). The high-resolution limit used for the search was typically 3 Å. By default, *HySS* was run multiple times on each data set using a different random seed each time and the solution with the highest correlation co­efficient between structure factors calculated from the heavy-atom model and the structure-factor differences or *F*_A_ values was kept. The correlation coefficient was also used, along with the number of sites found, to determine whether to continue searching. Normally, the search was carried out ten times unless the expected number of sites was found and a correlation of 0.3 was obtained. By default, if no solution was found with a correlation of at least 0.2 at a particular resolution, then up to two additional high-resolution limits were tested in steps of 1 Å (*e.g.* using a high-resolution limit of 3 Å followed if necessary by high-resolution limits of 4 and 5 Å.

#### 2.1.3. Phasing with *Phaser* and *SOLVE* and map evaluation

Each potential heavy-atom substructure found above (along with its inverse) was used to calculate phases with *Phaser* (for SAD phasing; McCoy *et al.*, 2004 ▶) or *SOLVE* (for MAD, SIR and MIR phasing; Terwilliger & Berendzen, 1996 ▶, 1997 ▶, 1999*a* ▶,*b* ▶). (In the examples shown in this work and in *PHENIX* versions up to v.1.3 the hand of the space group was fixed; later versions of *PHENIX* automatically invert chiral space groups when considering the inverse of the substructure.) The resulting phases and amplitudes of structure factors, along with weights (the figure of merit of phasing), were used to calculate experimental electron-density maps using a high-resolution limit of 2.5 Å (or lower if data were not available to this resolution). The high-resolution limit was applied in order to reduce the effects of variable resolution limits on the features of electron-density maps. These maps were evaluated with the measures of map quality described in this work and the overall Bayesian estimate of quality was used to rank solutions. In cases where two solutions have very similar heavy-atom parameters (r.m.s.d. among heavy-atom coordinates of less than 1/10 of the high-resolution limit of the data), only the solution with the higher estimate of quality was considered. The estimate of uncertainty in the map quality was used to identify solutions that might plausibly (5% possibility or greater) be the best solution and normally all such solutions were considered at each step. By default, up to three of the highest ranking solutions (six for MIR structures) for the heavy-atom substructure were used to calculate phases and weights at the full available resolution of the data and for density modification.

In the structure determinations carried out below for development of the map evaluation criteria, rankings were instead obtained using a *Z*-score procedure (Terwilliger & Berendzen, 1999*a* ▶,*b* ▶) based only on the skewness of the electron density (as defined below).

#### 2.1.4. Statistical density modification with *RESOLVE*

The experimental phases obtained above were used as a starting point for statistical density modification using *RESOLVE* (Terwilliger, 2000 ▶).

In statistical density modification with the *PHENIX Auto­Sol* wizard, a probabilistic estimate of the boundary between macromolecule and solvent is identified in two ways and that leading to the lower *R* factor in density modification is used. The first method (Wang, 1985 ▶) is based on the local r.m.s. density, smoothing the squared density using a sphere (Leslie, 1987 ▶) with a smoothing radius (*r*_smooth_) given by an empirically derived formula (chosen by optimizing parameters carrying out density modification using model data),
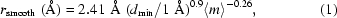
where *d*_min_ is the high-resolution limit of the data and 〈*m*〉 is the mean figure of merit of phasing. The second method for solvent-boundary identification uses a comparison of histograms of density based on model maps calculated with partially randomized phases with local histograms of density in the experimental map to assign a probability that each point in the map is part of the macromolecule or part of the solvent region. In both cases a probabilistic solvent boundary is obtained (Terwilliger, 1999 ▶).

Noncrystallographic symmetry (NCS) is used in density modification if it is detected based on the heavy-atom sub­structure and the presence of correlated density at NCS-related positions in the electron-density map (Terwilliger, 2002*a* ▶,*b* ▶). The value of *r*_smooth_ described above is used as a smoothing radius in a local correlation map to identify the region over which NCS holds (Vellieux *et al.*, 1995 ▶).

#### 2.1.5. Model building with *RESOLVE*

After density modification, the *PHENIX AutoSol* wizard carries out automated model building using a single cycle of building with the *PHENIX AutoBuild* wizard (Terwilliger *et al.*, 2008 ▶) or using rapid methods for building secondary structure of proteins and nucleic acids (T. Terwilliger, unpublished work). Initially, a secondary-structure-only model is built into each map. The correlation between a map calculated from the model and the density-modified map is then determined. If the value of the map–model correlation is less than a preset value (typically 0.35), then the building procedure is repeated with a standard cycle of building using the methods in the *PHENIX AutoBuild* wizard. If a map–model correlation of a given value (typically 0.20) or greater is obtained for at least one solution, then the top solution is identified as that with the highest value of the map–model correlation. If a lower map–model correlation is obtained, then the top solution is identified (see below) based on the Bayesian estimates of quality using the skewness of electron density (skew) and the correlation of local r.m.s. density (*r* ^2^_RMS_).

### 2.2. Evaluation of measures of map quality

A set of measures of map quality were applied to experimental maps (or structure-factor amplitudes, phases and weights) obtained from real but re-enacted structure determinations. Each of the structures considered had been determined previously, so that phases from a refined model could be used with measured amplitudes to calculate a model map to use as a standard. The ‘true’ quality of each map was taken to be the correlation with the corresponding standard map calculated at the same nominal resolution. Each measure of quality was applied to each map and the resulting scores were saved along with the corresponding ‘true’ quality. The structure-solution process was automatically carried out by the *PHENIX AutoSol* wizard and each experimentally phased map that was obtained during the structure-solution process was examined in this way. To reduce the number of near-duplicate solutions considered, all solutions for a structure that had nearly identical values of the map correlation to the standard map (within a range of ±0.0005 in map correlation) were considered to be the same and only the first was used in the analysis. For comparisons involving two possible enantiomers of a solution, the two enantiomers of a solution sometimes differed only slightly (*i.e.* the heavy-atom sub­structure was nearly centrosymmetric). In these analyses of enantiomeric pairs, only those that differed by an r.m.s.d. of at least 0.5 Å were considered.

For analysis of map quality, electron-density maps and structure factors were calculated using a high-resolution limit of 2.5 Å (if data were available to that resolution), as described above for the *PHENIX AutoSol* wizard. Before applying each of the measures of map quality, the experimental maps were normalized to a mean of zero and a variance of unity. They were then adjusted in two steps to reduce the contribution from high density at the coordinates of heavy-atom sites. (The high density at heavy-atom sites might otherwise lead to high values for the skewness, NCS correlation, contrast and possibly other measures.) Firstly, the electron density within a radius (*r*) of each heavy-atom site used in phasing (where *r* was given by twice the resolution of the data or 5 Å, whichever was greater) was limited to values less than or equal to twice the r.m.s. (2σ) of the map. Secondly, the electron density everywhere in the map was limited to values in the range −5σ to +5σ. This modified map is referred to below as the normalized truncated experimental electron-density map.

Weighted electron-density maps were calculated in the *PHENIX* environment (Adams *et al.*, 2002 ▶) using *RESOLVE* (Terwilliger, 2000 ▶) on a grid with a spacing of 1/3 of the high-resolution limit of the data or finer. Map correlations were obtained by calculating the correlation coefficient of a pair of maps at all the grid points in the asymmetric unit of the unit cell. Model–map correlations were calculated in the same way, except that one map was calculated from the model and an overall *B* factor (b_overall) was adjusted to maximize the correlation. This correlation was further maximized by adjusting a parameter (*r*_FFT_) representing the radius around atoms in the model to be included in FFT-based density calculations (typically about equal to the high-resolution limit of the data). For protein chains, an increment in isotropic thermal factors (beta_b) for each bond between side-chain atoms and the C^β^ atom was also applied to maximize the correlation.

### 2.3. Real-space map-quality measures

The measures of map quality used in this work are described in this and the following section and are summarized in Table 1 ▶.

#### 2.3.1. Skewness of electron density

The skewness (skew) of each normalized truncated map (as described in §[Sec sec2.1]2.1) was calculated using the relation
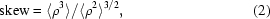
where the electron density (ρ) was calculated at all the grid points in the asymmetric unit. This quantity reflects the skewness of the density values in the map.

#### 2.3.2. Contrast of electron density

The contrast between the r.m.s. (root-mean-square) density in the solvent region and the r.m.s. density in the macromolecular region was calculated from the standard deviation of the local r.m.s. density over the entire asymmetric unit (Terwilliger & Berendzen, 1999*a* ▶; Sheldrick, 2002 ▶). The normalized truncated density described in §2.2 was first squared. The squared density was then smoothed by averaging all values within a moving sphere with radius (*r*) given by the larger of 6 Å or twice the high-resolution limit of the data. The standard deviation (*s*) of the smoothed squared density was then calculated. To compensate for the effect of the solvent fraction in the crystal (*f*) on the resulting value, the standard deviation (*s*) calculated above was multiplied by the factor [(1 − *f*)/*f*]^1/2^ to yield the contrast *c*, 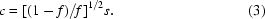
The correction factor [(1 − *f*)/*f*]^1/2^ was chosen because it leads to a value of 1 for the contrast for a map for which the entire solvent region has zero variance and the nonsolvent region has a constant and nonzero variance.

#### 2.3.3. Correlation of local r.m.s. density

The presence of contiguous flat solvent regions in a map was detected using the correlation coefficient of the smoothed squared electron density, calculated as described above, with the same quantity calculated using half the value of the smoothing radius, yielding the correlation of r.m.s. density, *r* ^2^_RMS_. In this way the local value of the r.m.s. density within a small local region (typically within a radius of 3 Å) is compared with the local r.m.s. density in a larger local region (typically within a radius of 6 Å). If there were a large contiguous solvent region and another large contiguous region containing the macromolecule, the local r.m.s. density in the small region would be expected to be highly correlated with the r.m.s. density in the larger region. On the other hand, if the ‘solvent’ region were broken up into many small flat regions, then this correlation would be expected to be smaller.

#### 2.3.4. Flatness of the solvent region

A normalized truncated electron-density map was partitioned between regions of solvent and macromolecule as described in §2.1.4. The r.m.s. electron density in the solvent region (r.m.s._SOLVENT_) and in the region of the macromolecule (r.m.s._PROT_) were then calculated. The flatness (*F*) of the solvent region was expressed as the difference between the two,
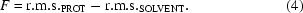

               

#### 2.3.5. Number of regions enclosing high density

A threshold of density (*t*) was found such that 5% of the volume of the asymmetric unit of the crystal had a density greater than this threshold *t*. All the grid points in the map above the threshold *t* were marked. The number of discrete regions (*N*_regions_) containing marked points was then counted. For this purpose, a discrete region was defined as a set of all marked grid points that can be connected by tracing from one adjacent marked grid point to another (including symmetry-related marked grid points). To partially compensate for the fact that lower resolution maps have fewer grid points, the number of regions was multiplied by the high-resolution limit of the data used to calculate the map (*d*_min_). To further compensate for the volume of the asymmetric unit containing the macromolecule, the number of regions was then divided by the fraction of the asymmetric unit that contains macromolecule (*f*) and the volume of the asymmetric unit (*V*) to yield the normalized number of regions per unit volume (*N*_r_), 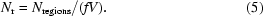

               

#### 2.3.6. Overlap of NCS-related density

If noncrystallographic symmetry was found in the heavy-atom substructure for a solution, then the map was examined for the presence of correlated density at NCS-related locations in the map (Cowtan & Main, 1998 ▶; Vellieux *et al.*, 1995 ▶). The overlap (*O*_NCS_) between density at NCS-related locations was used to evaluate noncrystallographic symmetry,
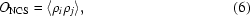
where ρ_*i*_ and ρ_*j*_ are density at NCS-related locations in the asymmetric unit and the average is either within a sphere with radius *r*_smooth_ (as described above for identifying the solvent boundary) or over a region within the asymmetric unit. The values of density ρ_*i*_ used were those from the normalized truncated map described above. The region where NCS applies was identified as a contiguous region in which the local mean of the overlap is at least *c*_MIN_, where this cutoff *c*_MIN_ was selected to yield a total volume occupied by all NCS copies that was approximately the same as the total volume (*f*) occupied by the macromolecule in the asymmetric unit (Terwilliger, 2002*a* ▶). For the purposes of evaluating a map, the mean value of the overlap of NCS density, *O*_NCS_, was calculated over this entire NCS region. If the value of the overlap found was less than *O*_MIN_ (typically, *O*_MIN_ = 0.3), the NCS was ignored.

### 2.4. Reciprocal-space map-quality measures

#### 2.4.1. *R* factor and phase correlation from statistical density modification

The amplitudes and phases of structure factors calculated using statistical density modification, but without including the experimental phase probabilities, can be compared with the observed amplitudes and experimental phases (Cowtan & Main, 1996 ▶; Terwilliger, 2001 ▶). These comparisons yield an *R* value (*R*_DENMOD_) for the amplitudes and a mean cosine of the phase difference (*m*_DENMOD_) for the phases.

#### 2.4.2. Figure of merit of phasing

The mean figure of merit of phasing (〈*m*〉) was used directly from *Phaser* (for SAD phasing calculations; McCoy *et al.*, 2004 ▶) or *SOLVE* (for MIR and MAD phasing calculations; Terwilliger & Berendzen, 1999*a* ▶,*b* ▶) as an estimate of the quality of a map.

#### 2.4.3. Density truncation (peak-picking)

The number of non-H atoms (*n*) in the asymmetric unit was roughly estimated from the fraction of the asymmetric unit that contains macromolecule (*f*) and the volume of the asymmetric unit (*V*) using an approximate average atomic volume *V*_o_ = 19 Å^3^ (Stroud & Fauman, 1995 ▶) using the relation *n* = *fV*/*V*_o_. The highest 3*n*/4 grid points in the asymmetric unit of the electron-density map were then identified and C atoms were placed at these grid points. A map was calculated from these C atoms and the correlation (*r* ^2^_TRUNCATION_) with the original map was obtained after adjusting an overall thermal factor to maximize this correlation.

### 2.5. Bayesian estimates of map quality

A simple Bayesian approach was used to create estimators of map quality based on one or more of the measures of map quality described in §§[Sec sec2.3]2.3 and [Sec sec2.4]2.4. For each measure (*e.g.* skew), the comparison of maps with the corresponding solved structures yielded a list of values of ‘true’ map correlation (*r* ^2^_MODEL_) and the measure of quality (*e.g.* skew). A two-dimensional histogram was created to represent the joint distribution *p*(*r* ^2^_MODEL_, skew). The distributions were sampled with 30 bins for each variable, with the range of allowed values of each ranging from −0.1 to 1.1. Any values obtained outside this range were put in the closest available bin. To compensate for the fact that insufficient data (1359) were present to generate an accurate value for all 900 bins, the values of *p*(*r* ^2^_MODEL_, skew) were smoothed using a Gaussian smoothing algorithm in which *p*(*r* ^2^_MODEL_, skew) was convoluted with a Gaussian function *G*(*r*) with a radius (σ) of three bins {*G*(*r*) ∝ exp[−(*u* ^2^ + *v* ^2^/(2σ^2^)]}, reducing the effective number of bins to about 100.

To estimate the value of map quality (*r* ^2^_MODEL_) from a new observation of the quality measure (skew), Bayes’ rule (Hamilton, 1964 ▶) was used,
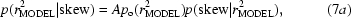
where the normalization factor *A* assures that the integrated probability for *r* ^2^_MODEL_ is unity and is given by
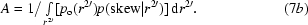
(7*a*) says that the (posterior) probability of a particular value of *r* ^2^_MODEL_, given the measurement skew, is the prior probability of *r* ^2^_MODEL_ [*p*_o_(*r* ^2^_MODEL_)] multiplied by the conditional probability [*p*(skew|*r* ^2^_MODEL_)] of measuring this value of skew given that *r* ^2^_MODEL_ is the correct value, divided by a normalization factor. We calculated the conditional probability *p*(skew|*r* ^2^_MODEL_) in (7*a* [Disp-formula fd7]) from the joint probability distribution *p*(*r* ^2^_MODEL_, skew) using the relation
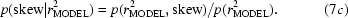
For the present work, we assume that the prior probability distribution *p*_o_(*r* ^2^_MODEL_) is uniform on [0, 1].

If several measures of map quality (*e.g.* skew and the contrast *c*) have been measured, then the estimates can be combined using the same approach:
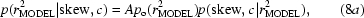

               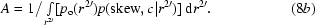
We approximate the probability distribution *p*(skew, *c*|*r* ^2^_MODEL_) as the product of the two two-dimensional conditional probabilities that we have estimated above, 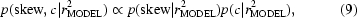
which amounts to assuming that the skewness and contrast *c* are conditionally independent for a given fixed *r* ^2^_MODEL_ value.

To obtain the estimated value and variance of *r* ^2^_MODEL_ given a set of observations of predictor variables (*e.g.* skew, *c*), we used the probability distribution given by (8*a*) and calculated the expectation value of 〈*r* ^2^_MODEL_〉, 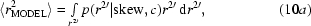

               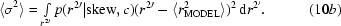
An improved estimate of the conditional probability distributions such as *p*(skew, *c*|*r* ^2^_MODEL_) could potentially be obtained by calculating the covariance of the variables skew and *c* for each fixed value of *r* ^2^_MODEL_ and assuming a normal distribution of skew and *c* for this fixed value of *r* ^2^_MODEL_. This formulation differs from that in (9) by including correlations between skew and *c* instead of assuming that they are zero and also through the assumption of normality in the distributions of skew and *c* for fixed *r* ^2^_MODEL_. Leaving out the fixed value of *r* ^2^_MODEL_ for clarity, representing the two-dimensional vector (skew, *c*) as **x** = (skew, *c*) and the mean values of skew and *c* for this value of *r* ^2^_MODEL_ as **u** = (〈skew〉, 〈*c*〉), we can write (Hamilton, 1964 ▶)
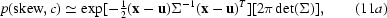
where Σ is the covariance matrix with elements σ_*ij*_ representing the variation of skew and *c* around their means 〈skew〉 and 〈*c*〉,
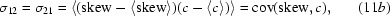

               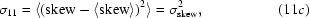

**Figure 1:**
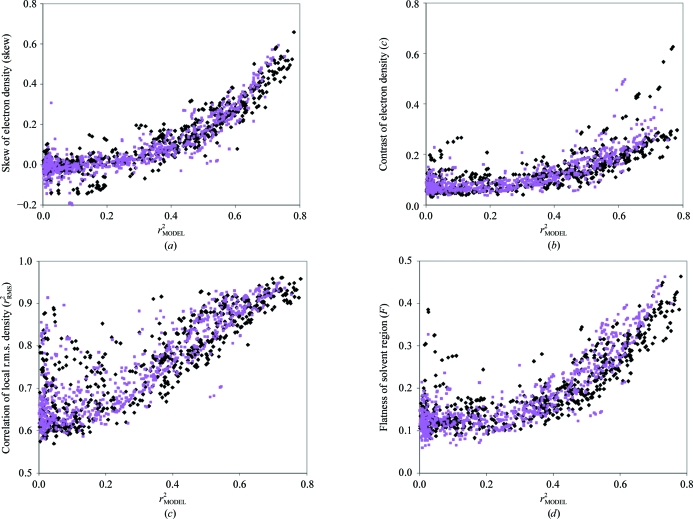

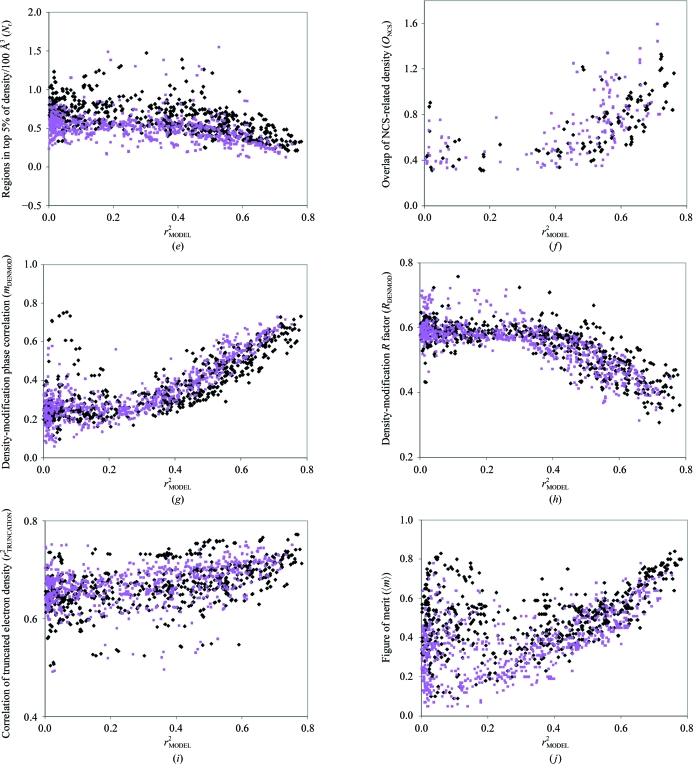
Measures of the quality of electron-density maps and structure factors. Measures of quality were calculated as described in the text for 1359 sets of structure factors and associated maps. Each measure is plotted with an abscissa equal to the correlation of density of the map with a map calculated from a final model (*r* ^2^_MODEL_). Measures based on structures determined at resolutions of 2 Å or higher are shown as black diamonds and those at resolutions lower than 2 Å are shown as purple squares. All measures of quality and the correlation with model density (*r* ^2^_MODEL_) were calculated at a resolution of 2.5 Å or the nominal resolution of the data, whichever is the lower. (*a*) Skewness of electron density. (*b*) Contrast of electron density. (*c*) Correlation of local r.m.s. density. (*d*) Flatness of solvent region. (*e*) Number of regions enclosing high density. (*f*) Overlap of NCS-related density. (*g*) Phase correlation from statistical density modification. (*h*) *R* factor from statistical density modification. (*i*) Density truncation. (*j*) Figure of merit of phasing.

               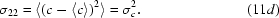
To test this approach we used the data described above, but grouped in bins of *r* ^2^_MODEL_. The observations in each bin of *r* ^2^_MODEL_ were analyzed using (11*a*)–(11*d*) [Disp-formula fd16] [Disp-formula fd17] [Disp-formula fd18] based on the values of the *N* predictor variables (skew, *c*…) for all the observations in that bin to obtain an approximation of the conditional probability distribution *p*(skew, *c*|*r* ^2^_MODEL_) for that bin. This set of approximations (one for each bin of *r* ^2^_MODEL_) was then used in (8) [Disp-formula fd11] to estimate *r* ^2^_MODEL_ for individual sets of observations of the *N* predictor variables. This approach gave correlations that were at most marginally improved over those obtained using estimates of the conditional probability distribution *p*(skew, *c*|*r* ^2^_MODEL_) based on (9). For example, using skew and correlation of local r.m.s. density (*r* ^2^_RMS_) as predictor variables and analyzing the same data shown in Table 3 (but without cross-validation), the overall correlation coefficient between the true values of *r* ^2^_MODEL_ and estimates obtained using (9) (in which independence of skew and *r* ^2^_RMS_ is assumed) was 0.925. Using (10) [Disp-formula fd14] [Disp-formula fd15] (assuming Gaussian distributions for skew and *r* ^2^_RMS_) and setting the covariance terms to zero (assuming independence of skew and *r* ^2^_RMS_) yielded a value of 0.926; the same analysis but including the covariance terms yielded a value of 0.927. As this approach did not significantly improve the correlation, it was not used. Fig. 1(*c*) suggests that the assumption of normality in the distributions of the predictor variables (*e.g.* skew and* r* ^2^_RMS_) for fixed *r* ^2^_MODEL_ is not well justified. This may partially explain the poor performance of this approach.

### 2.6. Structures and data used

Data from 47 structures in the *PHENIX* library of MAD, SAD and MIR data sets were used along with 246 MAD and SAD structures from the Joint Center for Structural Genomics (JCSG; http://www.jcs.org). The structures from the *PHENIX* library included 1029B (PDB code 1n0e; Chen *et al.*, 2004 ▶), 1038B (1lql; Choi *et al.*, 2003 ▶), 1063B (1lfp; Shin *et al.*, 2002 ▶), 1071B (1nf2; Shin, Roberts *et al.*, 2003 ▶), 1102B (1l2f; Shin, Nguyen *et al.*, 2003 ▶), 1167B (1s12; Shin *et al.*, 2005 ▶), aep-transaminase (1m32; Chen *et al.*, 2002 ▶), armadillo (3bct; Huber *et al.*, 1997 ▶), calmodulin (1exr; Wilson & Brunger, 2000 ▶), cobd (1kus; Cheong *et al.*, 2002 ▶), cp-synthase (1l1e; Huang *et al.*, 2002 ▶), cyanase (1dw9; Walsh *et al.*, 2000 ▶), epsin (1edu; Hyman *et al.*, 2000 ▶), flr (1bkj; Tanner *et al.*, 1996 ▶), fusion-complex (1sfc; Sutton *et al.*, 1998 ▶), gene-5 (1vqb; Skinner *et al.*, 1994 ▶), gere (1fse; Ducros *et al.*, 2001 ▶), gpatase (1ecf; Muchmore *et al.*, 1998 ▶), granulocyte (2gmf; Rozwarski *et al.*, 1996 ▶), groEL (1oel; Braig *et al.*, 1995 ▶), group2-intron (1kxk; Zhang & Doudna, 2002 ▶), hn-rnp (1ha1; Shamoo *et al.*, 1997 ▶), ic-lyase (1f61; Sharma *et al.*, 2000 ▶), insulin (2bn3; Nanao *et al.*, 2005 ▶), lysozyme (unpublished results; CSHL Macromolecular Crystallo­graphy Course), mbp (1ytt; Burling *et al.*, 1996 ▶), mev-kinase (1kkh; Yang *et al.*, 2002 ▶), myoglobin (A. Gonzales, personal communication), nsf-d2 (1nsf; Yu *et al.*, 1998 ▶), nsf-n (1qcs; Yu *et al.*, 1999 ▶), p32 (1p32; Jiang *et al.*, 1999 ▶), p9 (1bkb; Peat *et al.*, 1998 ▶), pdz (1kwa; Daniels *et al.*, 1998 ▶), penicillopepsin (3app; James & Sielecki, 1983 ▶), psd-95 (1jxm; Tavares *et al.*, 2001 ▶), qaprtase (1qpo; Sharma *et al.*, 1998 ▶), rab3a (1zbd; Ostermeier & Brunger, 1999 ▶), rh-dehalogenase (1bn7; Newman *et al.*, 1999 ▶), rnase-p (1nz0; Kazantsev *et al.*, 2003 ▶), rnase-s (1rge; Sevcik *et al.*, 1996 ▶), rop (1f4n; Willis *et al.*, 2000 ▶), s-hydrolase (1a7a; Turner *et al.*, 1998 ▶), sec17 (1qqe; Rice & Brunger, 1999 ▶), synapsin (1auv; Esser *et al.*, 1998 ▶), synaptotagmin (1dqv; Sutton *et al.*, 1999 ▶), tryparedoxin (1qk8; Alphey *et al.*, 1999 ▶), ut-synthase (1e8c; Gordon *et al.*, 2001 ▶) and vmp ( l8w; Eicken *et al.*, 2002 ▶).

The structures from the JCSG included PDB (Bernstein *et al.*, 1977 ▶; Berman *et al.*, 2000 ▶) entries 1o1x (Xu *et al.*, 2004 ▶), 1vjf, 1vjr, 1vk4, 1vk8, 1vk9, 1vkd, 1vkn, 1vl0, 1vl5, 1vli, 1vlo, 1vly, 1vm8, 1vmg, 1vmi, 1vp8, 1vpm, 1vpz (Rife *et al.*, 2005 ▶), 1vqr (Xu, Schwarzenbacher, McMullan *et al.*, 2006 ▶), 1vqs, 1vqy, 1vqz, 1vr0 (DiDonato *et al.*, 2006 ▶), 1vr3 (Xu, Schwarzenbacher, Krishna *et al.*, 2006 ▶), 1vr5, 1vr8 (Xu, Krishna *et al.*, 2006 ▶), 1vrm (Han *et al.*, 2006 ▶), 1z82, 1z85, 1zbt, 1zej, 1zh8, 1zko, 1ztc, 1zx8 (Jin *et al.*, 2006 ▶), 1zy9, 1zyb, 2a3n, 2aam, 2aml, 2ax3, 2b8n (Schwarzenbacher *et al.*, 2006 ▶), 2etd, 2ets, 2evr, 2f4i, 2f4l, 2fg0, 2fg9, 2fna, 2ftr, 2fup, 2fur, 2g0w, 2gb5, 2gc9, 2gf6, 2gfg, 2ghr (Zubieta *et al.*, 2007 ▶), 2gno, 2go7, 2gpi, 2gpj, 2grj, 2gvh, 2h1q, 2h1t, 2h9f, 2hcf, 2hh6, 2hhz, 2hi0, 2hq7, 2hq9, 2hr2, 2hsz, 2huh, 2hx1, 2hx5, 2hxv, 2i02, 2i8d, 2i9w, 2ig6, 2ii1, 2ilb, 2isb, 2it9, 2itb, 2nuj, 2o08, 2o2g, 2o2x, 2o2z, 2o3l, 2o62, 2oa2, 2oaf, 2oc6, 2od5, 2ogi, 2oh1, 2oh3, 2oik, 2ooj, 2ook, 2op5, 2opl, 2oqm, 2ord, 2osd, 2otm, 2ou3, 2ou5, 2ou6, 2own, 2oyo, 2ozg, 2ozj, 2p10, 2p1a, 2p7i, 2p8j, 2pbl, 2peb, 2pfw, 2pg4, 2pgc, 2pke, 2pn1, 2pq7, 2pr7, 2prr, 2prv, 2pv4, 2pv7, 2pwn, 2py6, 2pyq, 2pyx, 2q02, 2q04, 2q0t, 2q14, 2q3l, 2q78, 2q7x, 2q9k, 2q9r, 2qe6, 2qe9, 2qez, 2qg3, 2qhp, 2qj8, 2ql8, 2qml, 2qpx, 2qr6, 2qtp, 2qtq, 2qw5, 2qww, 2qwz, 2qyv, 2r01, 2r0x, 2r1i, 2r3b, 2r44, 2r4i, 2r9v, 2ra9, 2ras, 2rcc, 2rcd, 2rd9, 2rdc, 2re3, 2re7, 2rfp, 2rgq, 2rha, 2rhm, 2rij, 2ril, 2rkh, 3b5e, 3b5o, 3b77, 3b7f, 3b81, 3b8l, 3bb5, 3bb9, 3bcw, 3bdd and 3bde.

## 3. Results and discussion

### 3.1. Measures of map quality

A key goal of this work was to identify one or more quality measures of maps or of structure factors that are simple to calculate and that can yield accurate estimates of the qualities of the corresponding electron-density maps. Table 1 ▶ lists six measures of map quality examined here that are based on the features of the maps (real-space measures) and Table 2 ▶ lists four additional measures that depend on the structure factors and phases used to calculate maps. The measures were chosen to represent a range of possible measures that cover many important features of electron-density maps and structure factors.

To evaluate these measures of map quality, we carried out a re-analysis of data for 246 previously solved MAD, SAD and MIR structures, creating electron-density maps during the structure-determination process and analyzing them with each of the measures in Tables 1 ▶ and 2 ▶. As the structures are all known, the ‘true’ map quality for each map could be calculated as the correlation coefficient *r* ^2^_MODEL_ between each map and the corresponding map obtained using phases calculated from the refined model of the structure (after any necessary origin shifts are applied) using the *PHENIX* tool *phenix.get_cc_mtz_mtz*.

For each of the 246 data sets, the *PHENIX AutoSol* wizard was used to scale the data, calculate anomalous or isomorphous differences and identify potential heavy-atom solutions. As both hands of the heavy-atom substructure would normally be considered, at least two sets of heavy-atom solutions were generally obtained for each data set. Additionally, as MIR and MAD data sets have more than one set of anomalous or isomorphous differences, these data sets generally yielded additional heavy-atom solutions. Also for MIR and MAD structure determinations, difference Fourier analysis was used to generate even more heavy-atom solutions. Consequently, there were a total of 1359 heavy-atom solutions analyzed in this work even though there were only 246 data sets.

Figs. 1 ▶(*a*)–1 ▶(*j*) show the values of each measure plotted against *r* ^2^_MODEL_ for 1359 maps based on structures calculated from the MAD, SAD and MIR data listed in §[Sec sec2.6]2.6. The maps represent the phases obtained at several stages in structure determination. Some were calculated using heavy-atom solutions found from anomalous or isomorphous differences or from *F*_A_ values with *HySS* (Grosse-Kunstleve & Adams, 2003 ▶). Others were calculated using the corresponding substructures with inverted hand. Others were obtained from difference Fourier (MIR) and anomalous difference Fourier (MAD) analyses. In the case of MIR, a large number of additional solutions were obtained by combinations of partial solutions from different derivatives.

The general features of the plots in Fig. 1 ▶ are illustrated by a discussion of Fig. 1 ▶(*a*), which shows the skewness of electron density (skew) in experimental maps as a function of the true map quality *r* ^2^_MODEL_. In Fig. 1 ▶(*a*) the purple squares correspond to data sets with a nominal resolution lower than 2 Å and the black diamonds to data sets with resolutions of 2 Å or higher. (Note that the data for all these calculations were truncated at a resolution of 2.5 Å, so that most resolution-dependent differences are likely to be the consequence of data-set-dependent decreases of intensities with resolution rather than the resolution of the data.)

Fig. 1 ▶(*a*) shows that the skewness of the electron density depends strongly on the map quality, as represented by the correlation of the density in the map with that of a model map (*r* ^2^_MODEL_). The skewness is approximately zero for maps with a correlation in the range 0.0 < *r* ^2^_MODEL_ < 0.2. It increases slightly for maps with correlations in the range 0.2 < *r* ^2^_MODEL_ < 0.4 and then increases substantially for maps with higher correlations (*r* ^2^_MODEL_ > 0.4). The standard deviation of the values of the skewness is about 0.05–0.10 over most ranges of map correlation. For example, for values of map correlation with *r* ^2^_MODEL_ < 0.2 the mean skewness is −0.02 and the standard deviation is 0.07 and for values of map correlation with 0.4 < *r* ^2^_MODEL_ < 0.5 the mean skewness is 0.14 with a standard deviation of 0.06. For values of map correlation with 0.6 < *r* ^2^_MODEL_ < 0.7 the mean skewness is 0.38 with a standard deviation of 0.10. Another way to view these relationships is to note that the difference (0.16) in the mean values of the skewness between values of map correlation of *r* ^2^_MODEL_ < 0.2 and values of map correlation in the range 0.4 < *r* ^2^_MODEL_ < 0.5 is about twice the standard deviation of the skewness in either range. This means that the skewness can be expected to differentiate between maps with model correlations *r* ^2^_MODEL_ of zero and 0.4, but that it cannot differentiate them correctly all of the time. This can also be seen directly from Fig. 1 ▶(*a*), in which some of the values of skewness for maps with model correlations *r* ^2^_MODEL_ near 0.4 are lower than values for maps with near-zero values of *r* ^2^_MODEL_.

The maps represented in Fig. 1 ▶(*a*) that are based on high-resolution data sets (<2 Å) have values of skewness that are similar to those of lower resolution data sets. This similarity is most likely to reflect the fact that all the data in these calculations were truncated at a resolution of 2.5 Å.

Several of the other nine measures of map quality examined have relationships to model map correlation similar to those described above for the skewness. The contrast (*c*; Fig. 1 ▶ *b*), correlation of local r.m.s. density (*r* ^2^_RMS_; Fig. 1 ▶ *c*) and flatness of the solvent region (*F*; Fig. 1 ▶ *d*) in particular show very similar behaviour, except that none of these discriminate as well as the skewness between maps of moderate quality (correlations *r* ^2^_MODEL_ near 0.4) and those of very low quality with correlations near zero. These three measures are all related as they all are based on the presence of solvent and nonsolvent regions in the crystal. However, the calculations differ in that the con­trast (*c*) does not require knowledge of the solvent boundary while the flatness (*F*) does. Additionally, the correlation of local r.m.s. density reflects the contiguous nature of the solvent region while the contrast (*c*) and flatness (*F*) reflect the presence of a solvent region, whether contiguous or not.

A somewhat different behavior is shown by the number of contiguous regions (*N*_r_) required to enclose the highest 5% of density in a map (Fig. 1 ▶ *e*). This measure decreases with increasing map quality, but only slightly, so that it is not a strong discriminator between maps of low and moderate quality.

The overlap of NCS-related density (Fig. 1 ▶ *f*) is a measure which, as implemented here, only applies to maps where NCS can be identified from the symmetry present in the heavy-atom sites. It is therefore different from the measures discussed so far and cannot be used as a general measure of map quality. It is nevertheless useful in differentiating between maps with very high model map correlations (*r* ^2^_MODEL_) and those that have lower model map correlation.

Figs. 1 ▶(*g*) and 1 ▶(*h*) show the phase correlations (*m*_DENMOD_) and *R* factors (*R*_DENMOD_) obtained from the first cycle of statistical density modification using the same structure factors, phases and weights that were used to calculate the electron-density maps analyzed in Figs. 1 ▶(*a*)–1 ▶(*f*). In the first cycle of statistical density modification with *RESOLVE* (Terwilliger, 2000 ▶), estimates of the phase and amplitude of a reflection *k* were obtained using only information from all the other reflections in the data set. The amplitude and phase for reflection *k* from the density-modification procedure can then be compared with the experimentally observed amplitude and the ‘experimental’ phase (derived using isomorphous or anomalous differences) to yield an *R* factor for density modification (*R*_DENMOD_) and a mean cosine of the phase difference (*m*_DENMOD_). Fig. 1 ▶(*g*) shows that, as expected, the *R* factor for density modification decreases with increasing map quality, while Fig. 1 ▶(*h*) shows that the phase correlation increases over the same range.

Fig. 1 ▶(*i*) shows that the correlation of pseudo-maps calculated using dummy atoms placed at the highest peaks in a map with their corresponding original maps (*r* ^2^_TRUNCATION_) is weakly related to the quality of the map. It seems possible that more sophisticated methods of map skeletonization (Baker *et al.*, 1993 ▶) might be more useful in map evaluation than our simple measure.

Finally, Fig. 1 ▶(*j*) shows that the mean figure of merit of phasing (〈*m*〉) is related to the quality of the map, but that there are many maps with very low correlation to the corresponding model maps that nevertheless have high mean figures of merit. This relationship can be understood by con­sidering that the figures of merit of phasing of two maps that are calculated using the same data but opposite enantiomers of the heavy-atom substructure are normally identical for SAD phasing if all the anomalous scatterers are of the same type. Typically, one of these maps may have a high correlation to the model map while the other may have a very low correlation.

Overall, Fig. 1 ▶ shows that several measures of map quality based on different features of the map and on the structure factors and phases leading to the map have strong relationships to the quality of the electron-density map, with the skewness of electron density clearly being one of the best indicators of map quality.

**Figure 2:**
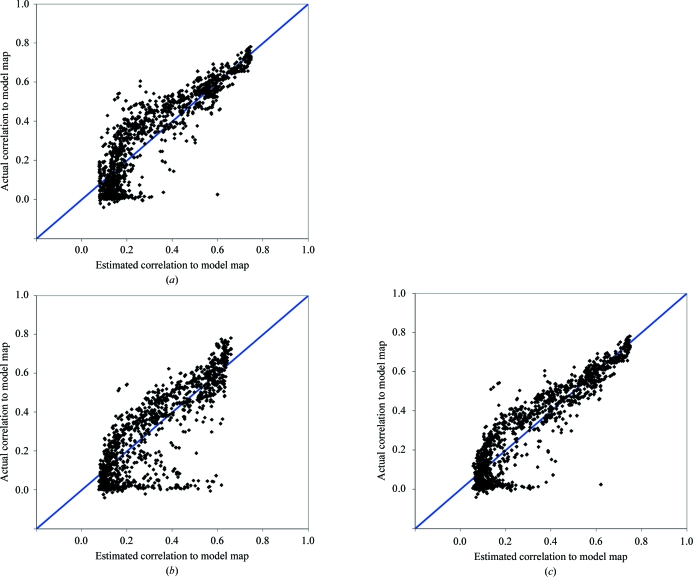
Comparisons of cross-validated estimates of map quality with actual map quality. Measures of map quality as shown in Fig. 1 ▶ were used in (7*a*) and (8*a*) to estimate overall map quality. The calculations were carried out one data set at a time. For each data set, joint probability distributions of each measure of quality and true quality [*e.g. p*(skew, *r* ^2^_MODEL_)] were calculated excluding data from all solutions for that structure. These cross-validated joint probability distributions were used in (7*a*) and (8*a*) to estimate map quality using the measures of quality for each map associated with that data set. In each case, the true map quality (*r* ^2^_MODEL_) is plotted as a function of the Bayesian estimates of map quality. (*a*) Estimates of map quality using the skewness of electron density in (7*a*). (*b*) Estimates using the correlation of local r.m.s. density in (7*a*). (*c*) Estimates using the skewness and correlation of local r.m.s. density in (8*a*).

In Fig. 1 ▶(*a*) there is one point at (*r* ^2^_MODEL_ = 0.03, skew = 0.31) that is quite far from all the others, with a value of the skewness that is far greater than all the other points with very small values of *r* ^2^_MODEL_. This point corresponds to a heavy-atom solution found during the analysis of data from PDB entry 2re3 which yields an electron-density map that is incorrect but not at all random. The crystal has translational noncrystallographic symmetry and the electron density in the electron-density map for this solution is offset from that the correct map by an origin shift that is noncrystallographic. Consequently, our analysis of the two maps, which only allows crystallographic translational offsets, shows a near-zero correlation of the maps despite considerable similarity (a correlation of 0.73 when offset). We note that the translation involved does correspond to a real difference: if the coordinates of PDB entry 2re3 are shifted by this translation (0, 0.735, 0) in space group *P*4_3_2_1_2 the amplitudes of the structure factors do change and the *R* factor based on experimental amplitudes for the model in this position is 0.53, compared with a value of 0.23 for the deposited model. Note that this solution also appears in Fig. 2 ▶(*a*) at the position (0.60, 0.03) and in Fig. 2 ▶(*b*) at the position (0.62, 0.03) where it is again an outlier.

### 3.2. Estimation of map quality using features of the map and of the structure factors used to calculate the map

Fig. 1 ▶ showed that each of the six different features of electron-density maps and the four characteristics of structure factors we examined depend in some way on the quality of the corresponding map. We used the Bayesian approach described in §[Sec sec2.5]2.5 to use this information to estimate map quality from these ten features. The general idea of this approach is very simple. Imagine that a particular map has been examined, yielding a value of the skewness of electron density of 0.20. Considering the plot in Fig. 1 ▶(*a*), it is reasonable to conclude that this map is very likely to have a correlation (*r* ^2^_MODEL_) with the corresponding model map in the range 0.4 < *r* ^2^_MODEL_ < 0.6, because nearly all examples in Fig. 1 ▶(*a*) with a skewness of about 0.20 are in this range. Equation 7(*a*) is a mathematical way to make this statement. Equation 8(*a*) is a similar statement, except that it includes more than one measure of map quality. As described in §[Sec sec2.5]2.5, we assume here that the various measures of map quality (skewness, contrast *etc*.) are independent. This allows the simple calculation in (8*a*) to be used to estimate *r* ^2^_MODEL_ from several measures of map quality.

Fig. 2 ▶(*a*) shows the results of using (7*a*) to estimate *r* ^2^_MODEL_ from the skewness of electron density. In Fig. 2 ▶(*a*) the abscissa is the Bayesian estimate of *r* ^2^_MODEL_ using the skewness of electron density and the ordinate is the true value of *r* ^2^_MODEL_. To ensure that the parameters in the Bayesian estimator did not contain information on the specific cases being tested, a cross-validation procedure was used in which all solutions for the structure being examined were excluded when con­structing the Bayesian estimators. Fig. 2 ▶(*a*) shows that in cases where the true value of *r* ^2^_MODEL_ is in the range 0.0 < *r* ^2^_MODEL_ < 0.2, the estimates of *r* ^2^_MODEL_ all have very similar values of about 0.1. This can be understood from Fig. 1 ▶(*a*), in which the skewness is seen to be insensitive to values of *r* ^2^_MODEL_ in this range. The Bayesian estimates of *r* ^2^_MODEL_ for low values of skewness are all close to the midpoint of this range, as they are simply the average of plausible values of *r* ^2^_MODEL_ given the observation of the value of the skewness. For higher values of *r* ^2^_MODEL_, the estimates of *r* ^2^_MODEL_ are closer to the true values. Overall, the correlation coefficient between the Bayesian estimates and the true values of *r* ^2^_MODEL_ is 0.90 and the r.m.s. error in prediction of *r* ^2^_MODEL_ is 0.10. As a check on our procedures, we note that the mean uncertainty estimates for *r* ^2^_MODEL_ obtained from the Bayesian procedure was 0.11, which is quite similar to the actual r.m.s. error in prediction of *r* ^2^_MODEL_ of 0.10.

Table 3 ▶ summarizes the accuracy of the Bayesian estimates of map quality based on each of the measures described in Tables 1 ▶ and 2 ▶ (with the exception of the overlap of NCS density, which is not included because it does not apply to most of the maps in our tests). For each measure, Table 3 ▶ lists the values of the correlation coefficient of the Bayesian estimates and the true map quality (*r* ^2^_MODEL_) along with the r.m.s. prediction error in *r* ^2^_MODEL_. Overall, the skewness of electron density, with a correlation coefficient between Bayesian estimates and true values of *r* ^2^_MODEL_ of 0.90, is the most reliable indicator of map quality, with the correlation of local r.m.s. density being the next best (correlation of 0.85) and with contrast, flatness of solvent region and density-modification phase correlations and *R* factor giving only slightly poorer predictions of *r* ^2^_MODEL_, with correlations in the range 0.75–0.80.

To identify an optimal combination of measures for estimation of map quality, we began with the best single measure (skew) and used (9) to combine information from each of the other measures. The measure giving the best prediction of *r* ^2^_MODEL_ in combination with the skewness of electron density was the correlation of local r.m.s. density (*r* ^2^_RMS_; Table 3 ▶). Fig. 2 ▶(*b*) shows how the estimates of map quality obtained using just the correlation of r.m.s. electron density compare with actual map quality and Fig. 2 ▶(*c*) shows estimates based on both skewness and correlation of r.m.s. electron density. The correlation of r.m.s. density was the next-best single pre­dictor after skew; in addition, the correlation of prediction errors from these two variables was relatively low (0.61; Table 4 ▶). The assumptions in (9) are therefore relatively well justified and it is not surprising that the resulting estimator is improved over that using just the skewness of electron density. This process was continued but no further improvement was obtained in the Bayesian estimator. The optimized combination of measures based on skewness and correlation of local r.m.s. density yielded a correlation coefficient between the Bayesian estimates and true values of *r* ^2^_MODEL_ of 0.92 and an r.m.s. prediction error of 0.09 (Table 3 ▶ and Fig. 2 ▶ *c*).

### 3.3. Identification of the hand of heavy-atom substructures using measures of map quality

**Figure 3:**
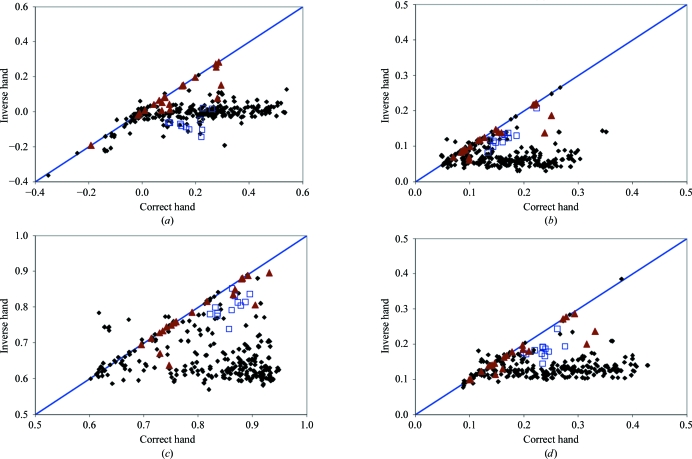

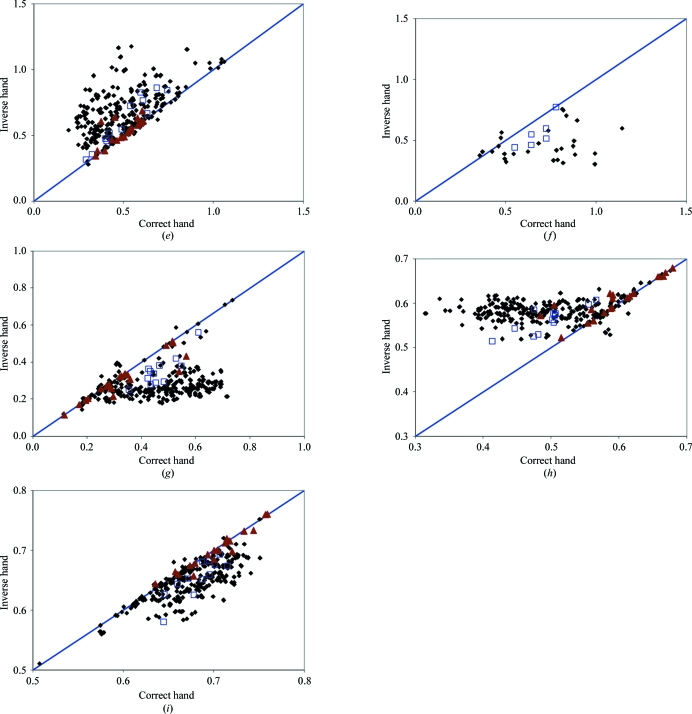
Comparisons of measures of map quality for pairs of maps based on enantiomorphic heavy-atom substructures. For structures in nonchiral space groups, all pairs of solutions derived from enantiomorphic pairs of heavy-atom substructures were selected. The member of the pair leading to the map with the higher correlation coefficient to the corresponding model map was identified as the ‘correct’ hand and the other as the ‘inverse’ hand. The value of each measure of map quality for the correct hand is plotted as the abscissa in each plot and the value of the measure for the corresponding inverse hand is the ordinate. Maps based on MAD data are represented as black diamonds, those from MIR data (all maps examined in this figure are from single derivatives) are represented as red triangles and those from SAD data are represented as blue squares. (*a*) Skewness of electron density. (*b*) Contrast of electron density. (*c*) Correlation of local r.m.s. density. (*d*) Flatness of solvent region. (*e*) Number of regions enclosing high density. (*f*) Overlap of NCS-related density. (*g*) Phase correlation from statistical density modification. (*h*) *R* factor from statistical density modification. (*i*) Density truncation.

A particularly important application of measures of map quality is the identification of the hand of heavy-atom sub­structures. The hand of the heavy-atom substructure cannot normally be identified directly during substructure determination by direct methods such as the *HySS* procedure (Grosse-Kunstleve & Adams, 2003 ▶) used here. Consequently, some procedure is needed for identifying which hand of the heavy-atom substructure is correct. Figs. 3 ▶(*a*)–3 ▶(*i*) compare the values obtained for nine measures of map quality based on 353 pairs of heavy-atom substructures with correct and inverted handedness from the 186 data sets in this work for which the space group was not chiral (structures with chiral space groups were excluded so the hand of the space group could be fixed in this analysis). The mean figure of merit of phasing is not shown because it is essentially identical for the two hands of the substructure in all the cases examined. The 706 maps represented by these 353 pairs are a subset of the 1359 maps used in the calculations shown in Fig. 1 ▶.

It is somewhat remarkable that these nine measures of map quality all give very good discrimination between the correct and incorrect hands of heavy-atom substructures (Fig. 3 ▶ and Table 5 ▶), even though they are not all so useful in estimating the absolute quality of maps (Table 3 ▶). The best discrimination between correct and incorrect hands is obtained with the skewness of electron density (Fig. 3 ▶ *a*), as expected from the high correlation of estimates of map quality based on skewness with actual map quality (Table 3 ▶). Using the skewness of electron density to make decisions on handedness (Fig. 3 ▶ *a*), 98% of decisions (in cases where the quality of the maps for the two hands differs by at least 0.05) would correctly identify the map with the higher quality (Table 5 ▶). Note that for SIR or MIR data without anomalous differences none of these techniques can identify the correct hand because the inverse hand of the heavy atoms leads to a map that has inverse chirality but is otherwise identical. A similar argument would partially apply in cases where an anomalous signal is present but is weak. This situation is presumably the cause of the large number of MIR-derived points near the diagonal of the panels in Fig. 3 ▶.

### 3.4. Identification of the highest quality density-modified map for a structure

The scoring procedures described above are based on an analysis of the phases and structure-factor amplitudes corresponding to an experimental electron-density map. Prior to final map interpretation, however, the experimentally determined phases of structure factors are normally optimized by density modification (Wang, 1985 ▶). Several additional parameters are required for density modification, including identification of noncrystallographic symmetry (if any), solvent content and the solvent region. It seemed possible that these parameters might not always be chosen optimally and the best experimental maps might not always lead to the best density-modified maps. Consequently, some additional method of scoring the density-modified maps might be useful.

To investigate this possibility, we carried out structure determination with the data sets used in Fig. 1 ▶, this time with default parameters in the *PHENIX AutoSol* wizard including Bayesian estimates of experimental map quality based on the skewness of electron density (skew) and the correlation of local r.m.s. density (*r* ^2^_RMS_). For each structure, the final steps were to carry out density modification with *RESOLVE* (Terwilliger, 2000 ▶) on the top-ranked solution or solutions and then to build a preliminary atomic model. In cases where there was one solution that was much better than all others (see §[Sec sec2]2), then only that solution was used in density modification. However, in most cases there were multiple solutions with similar Bayesian estimates of quality and up to three (MAD, SAD) or six (MIR) of these were used in density modification.

**Figure 4:**
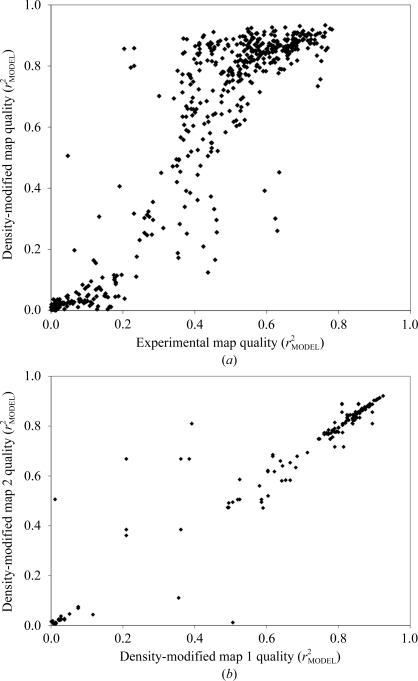
Map qualities of density-modified maps. (*a*) Qualities of density-modified maps as a function of the qualities of the corresponding experimental maps. (*b*) Comparison of qualities of pairs of density-modified maps for the same structure derived from experimental maps of similar quality (see text).

Fig. 4 ▶(*a*) shows the relationship between the qualities of experimental maps and the qualities of the corresponding density-modified maps for 569 experimental maps for 260 data sets. For experimental maps of high quality (correlation with model map over 0.6), the quality of the density-modified map is generally (but not always) very high, typically ranging from 0.75 to 0.90. For very poor experimental maps (correlation with model map of less than 0.2) the density-modified maps were also uniformly poor (typical map correlation of 0–0.1). On the other hand, for experimental maps of moderate quality (map correlation between 0.2 and 0.5) the quality of the density-modified maps vary over a wide range (from about 0.1 to about 0.9).

Much of the variability in density modification for experimental maps of moderate quality illustrated in Fig. 4 ▶(*a*) could arise from the intrinsic differences in solvent content, non­crystallographic symmetry, type of experiment and resolution between the different structures. To examine this, we have plotted in Fig. 4 ▶(*b*) the true map qualities of density-modified maps for all 176 pairs of solutions from Fig. 4 ▶(*a*) that are from the same structure, use the same number of non-crystallo­graphic symmetry operators (if any) in density modification and have values of true experimental map correlation within 0.05 of each other. In Fig. 4 ▶(*b*) each point corresponds to one pair of solutions. The abscissa is the value of density-modified map quality for the solution with the higher value of experimental map quality and the ordinate is the density-modified map quality for the solution with lower experimental map quality. Each member of such a pair has identical solvent content, resolution, actual noncrystallographic symmetry, number of noncrystallographic symmetry operators identified and experiment type and differs only slightly in true experimental map quality. Fig. 4 ▶(*b*) shows that when all these factors are controlled the quality of pairs of density-modified maps is very similar in most cases, but substantial differences in the qualities of the density-modified maps remain in some cases.

The remaining variation in effects of density modification illustrated in Fig. 4 ▶(*b*) suggests that it might be useful to carry out a final ranking of solutions based on a measure of quality of the corresponding density-modified maps. We used the map–model correlation between density-modified maps and the preliminary atomic models built with the *PHENIX AutoSol* wizard as such a measure of quality. Table 6 ▶ shows the utility of this map–model correlation in identifying the solution with the best density-modified map for each of the 149 structures used in Fig. 4 ▶(*a*) in which there was more than one solution tested by density modification and model building and in which the model-building process yielded a model with a model–map correlation of at least 0.20.

The first row in Table 6 ▶ provides a background for this analysis by considering the use of our Bayesian estimates of experimental map quality to identify the best solutions. In Table 6 ▶ experimental map quality and density-modified map quality are examined separately. Using the Bayesian estimates (which are based on the experimental maps), the best experimental map for a particular structure could be identified 91% of the time. The worst error in identification of the best experimental map corresponded to a difference in map correlation of 0.29. Next, density-modified maps were examined. The solution with the highest Bayesian estimate of experimental map quality led to the best density-modified map in 88% of cases; however, the worst error in identification of the best density-modified map corresponded to a very large difference in map correlation of 0.58.

Using the map–model correlation for the model built into the density-modified maps in decision-making the situation is reversed, with the best experimental map identified only 87% of the time and the best density-modified map identified 92% of the time. Further, the density-modified map yielding the highest map–model correlation was never worse than the very best density-modified map obtained by more than a difference in correlation of 0.26, showing that the model–map correlation is a useful criterion for final ranking of solutions. Overall, Table 6 ▶ indicates that model–map correlation is an improvement over Bayesian estimates of experimental map quality for the identification of the best density-modified map.

### 3.5. Using the *PHENIX AutoSol* wizard to redetermine structures from the *PHENIX* structure library

To test the overall utility of the Bayesian estimates of map quality in the overall context of structure determination, we carried out automated structure determinations on all 48 MAD, SAD and MIR structures in the *PHENIX* structure library with the *PHENIX AutoSol* wizard. The structures in this library range from relatively straightforward cases of SAD and MAD structure determination to considerably more complex cases that involved combinations of SAD or MAD with MIR and difficult-to-solve heavy-atom substructures. In the tests carried out here, only one source of phase information was used for each structure (*i.e.* MAD, SAD or MIR), except in the case of the fusion-complex structure (PDB code 1sfc; Sutton *et al.*, 1998 ▶), in which SAD and SIR data were combined.

**Figure 5:**
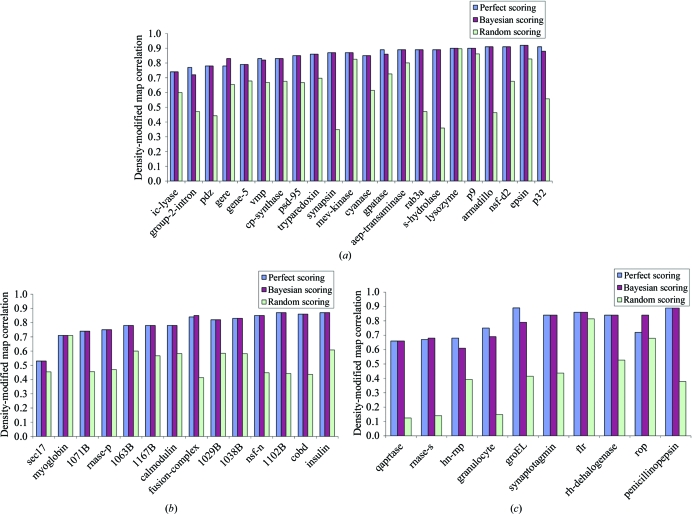
Comparison of quality of density-modified maps obtained using the skewness of electron density and correlation of local r.m.s. density for scoring with those obtained using the true map quality (correlation to the corresponding model map) for scoring. See text for details. The light blue bars labeled ‘Perfect scoring’ correspond to running the *PHENIX AutoSol* wizard and using the actual experimental map quality to make decisions at each step prior to obtaining density-modified phases and using the actual density-modified map quality to make the final choice of solution. The dark maroon bars labeled ‘Bayesian scoring’ correspond to using the Bayesian scores for experimental maps based on the skewness of electron density and correlation of local r.m.s. density and using the model–map correlation to choose the final density-modified solution. The light green bars labeled ‘Random scoring’ correspond to using random scores to make decisions about experimental map quality and model–map correlation to choose the final solution. Each ‘random scoring’ value is the average of ten separate runs of *PHENIX AutoSol* wizard carried out with differing random seeds. Note that the ‘perfect scoring’ method does not necessarily lead to the best final map. For example, an experimental map that is not the best one but is chosen by another scoring method could adventitiously yield additional sites that lead to a better final solution. (*a*) Structures determined using MAD. Structures shown are aep-transaminase (PDB code 1m32; Chen *et al.*, 2002 ▶), armadillo (3bct; Huber *et al.*, 1997 ▶), cobd (1kus; Cheong *et al.*, 2002 ▶), cp-synthase (1l1e; Huang *et al.*, 2002 ▶), cyanase (1dw9; Walsh *et al.*, 2000 ▶), epsin (1edu; Hyman *et al.*, 2000 ▶), gene-5 (1vqb; Skinner *et al.*, 1994 ▶), gere (1fse; Ducros *et al.*, 2001 ▶), gpatase (1ecf; Muchmore *et al.*, 1998 ▶), group2-intron (1kxk; Zhang & Doudna, 2002 ▶), ic-lyase (1f61; Sharma *et al.*, 2000 ▶), lysozyme (unpublished results; CSHL Macromolecular Crystallography Course), mbp (1ytt; Burling *et al.*, 1996 ▶), mev-kinase (1kkh; Yang *et al.*, 2002 ▶), nsf-d2 (1nsf; Yu *et al.*, 1998 ▶), p32 (1p32; Jiang *et al.*, 1999 ▶), p9 (1bkb; Peat *et al.*, 1998 ▶), pdz (1kwa; Daniels *et al.*, 1998 ▶), psd-95 (1jxm; Tavares *et al.*, 2001 ▶), rab3a (1zbd; Ostermeier & Brunger, 1999 ▶), s-hydrolase (1a7a; Turner *et al.*, 1998 ▶), synapsin (1auv; Esser *et al.*, 1998 ▶), tryparedoxin (1qk8; Alphey *et al.*, 1999 ▶) and vmp (1l8w; Eicken *et al.*, 2002 ▶) (*b*) Structures determined using SAD: 1029B (1n0e; Chen *et al.*, 2004 ▶), 1038B (1lql; Choi *et al.*, 2003 ▶), 1063B (1lfp; Shin *et al.*, 2002 ▶), 1071B (1nf2; Shin, Roberts *et al.*, 2003 ▶), 1102B (1l2f; Shin, Nguyen *et al.*, 2003 ▶), 1167B (1s12; Shin *et al.*, 2005 ▶), rnase-p (1nz0; Kazantsev *et al.*, 2003 ▶), calmodulin (1exr; Wilson & Brunger, 2000 ▶), fusion-complex (1sfc; Sutton *et al.*, 1998 ▶), insulin (2bn3; Nanao *et al.*, 2005 ▶), myoglobin (A. Gonzales, personal communication), nsf-n (1qcs; Yu *et al.*, 1999 ▶), sec17 (1qqe; Rice & Brunger, 1999 ▶) and ut-synthase (1e8c; Gordon *et al.*, 2001 ▶). Note that fusion-complex was solved with SAD plus SIR. (*c*) Structures determined using MIR: flr (1bkj; Tanner *et al.*, 1996 ▶), granulocyte (2gmf; Rozwarski *et al.*, 1996 ▶), groEL (1oel; Braig *et al.*, 1995 ▶), hn-rnp (1ha1; Shamoo *et al.*, 1997 ▶), penicillopepsin (3app; James & Sielecki, 1983 ▶), qaprtase (1qpo; Sharma *et al.*, 1998 ▶), rh-dehalogenase (1bn7; Newman *et al.*, 1999 ▶), rnase-s (1rge; Sevcik *et al.*, 1996 ▶), rop (1f4n; Willis *et al.*, 2000 ▶) and synaptotagmin (1dqv; Sutton *et al.*, 1999 ▶).

To evaluate the overall contribution of the Bayesian scoring approach described here to structure solution, we compared the qualities of the final density-modified maps obtained with the *PHENIX AutoSol* wizard using each of three different methods of making decisions during the heavy-atom solution and phasing steps of structure determination. The first method (‘perfect scoring’) was to use the actual correlation coefficient of each experimental map with that of the corresponding idealized map (using phases from a refined model) to decide which map was best during structure solution. Once density-modified maps had been calculated, the correlations of those maps with the idealized map were used for the final ranking. The second method (‘Bayesian scoring’) was to use the Bayesian estimates based on the combination of the skewness of electron density and the correlation of local r.m.s. density for decision-making during structure solution. Once density-modified maps had been calculated, a model was built and the correlation between this model and the density-modified map was used for final ranking. The third method (‘random scoring’) was to use random scores for decision-making during structure solution and then to use the model–map correlation for the final ranking. Fig. 5 ▶(*a*) illustrates these comparisons for MAD structure determinations, Fig. 5 ▶(*b*) illustrates them for SAD structure determinations and Fig. 5 ▶(*c*) for MIR structure determinations.

For MAD, SAD and MIR structure determinations the decision-making procedure using Bayesian estimates of experimental map quality and model–map correlations as estimates of density-modified map quality led to density-modified electron-density maps that were very similar in quality to those obtained using the decision-making process based on actual map quality (Fig. 5 ▶). This indicates that the quality of final density-modified maps produced by the *PHENIX AutoSol* wizard are essentially as good as they can be with any decision-making system, given the algorithms and parameters used to find heavy-atom sites and to carry out phasing, density modification and model building in the wizard.

In addition to the ‘perfect scoring’ and ‘Bayesian scoring’ approaches shown in Fig. 5 ▶, the figure includes density-modified map quality for solutions obtained using random scores for experimental maps (but still using model–map correlation to evaluate final density-modified maps). Each ‘random scoring’ value is the average of ten runs with differing random seeds, so they represent an average value of the quality of final maps obtained with random scoring of experimental maps. The quality of these maps is generally lower than that of those obtained with either of the other two methods, showing that the scoring is contributing important information to the structure-determination process.

Although Fig. 5 ▶ indicates that the quality of the final maps obtained with the *PHENIX AutoSol* wizard are essentially as good as they can be with the structure-solution algorithms in the wizard, it is likely that the number of solutions that need to be examined at each stage in structure determination could be lowered if improved estimates of experimental map quality were available. The default parameters in the *PHENIX AutoSol* wizard defining the number of solutions to keep at each stage were chosen to be large enough that the best solution was generally in the set that was considered at each stage using the 48 MAD, SAD and MIR data sets examined in Fig. 5 ▶. If improved scoring methods are developed, then a systematic re-examination of these default parameters would probably be useful. In the meantime, modifying these parameters to include larger or smaller numbers of solutions at each stage may be useful in cases that are more challenging or that are more straightforward, respectively.

**Figure 6:**
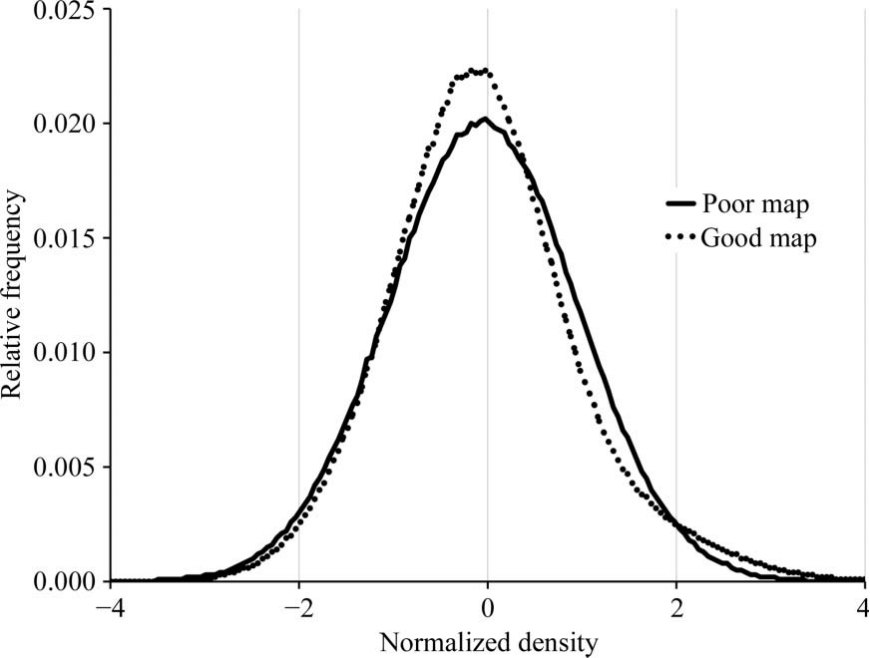
Histograms of density corresponding to a poor map (dotted lines, correlation to model map of 0.04) and to a good map (solid lines, correlation to model map of 0.66). See text for details.

The skewness of electron-density values in an electron-density map has been recognized for some time as a potential indicator of the quality of the map (Podjarny, 1976 ▶; Lunin, 1993 ▶). As the skewness of a map is not a familiar quantity to most crystallographers, we illustrate it for ‘poor’ and ‘good’ experimental electron-density maps. Both maps were based on experimental data for aep-transaminase (PDB code 1m32; Chen *et al.*, 2002 ▶) and were obtained during the course of automated analysis of this data with the *PHENIX AutoSol* wizard. The poor map was calculated using an incorrect set of heavy-atom sites and the good map was calculated using a largely correct set of heavy-atom sites. Fig. 6 ▶ shows histograms of the number of grid points in each map with various values of electron density. The *x* axis in Fig. 6 ▶ corresponds to electron density in a map normalized to the r.m.s. in the map after subtracting the mean of the map from all values. The dotted lines in Fig. 6 ▶ illustrate the fraction of grid points in the poor map that correspond to each value of normalized electron density. It may be seen that this histogram of densities from a poor map has a very nearly Gaussian shape. This poor map had a skewness of 0.004 and its correlation to a map based on the refined model of the structure was 0.04. In contrast, the solid lines in Fig. 6 ▶ illustrate the fraction of grid points in the good map corresponding to various values of electron density. This histogram differs from that derived from the poor map in that it is not symmetrical. The peak is slightly negative of the origin and it has a distinct tail on the positive side of the peak. This good map had a skewness of 0.4 and its correlation to the map based on the refined model was 0.66. Note that the differences in shapes of the histograms based on poor or good maps can be rather small, as in Fig. 6 ▶. Nevertheless, the skewness can usually be estimated very accurately because there are typically tens of thousands of grid points in the maps, so that the shapes of the histograms are very precisely defined.

## 4. Conclusions

Each of the ten measures of the quality of experimental electron-density maps evaluated here has some utility in estimating the true quality of these maps. These measures of map quality reflect a wide range of characteristics (Tables 1 ▶ and 2 ▶) ranging from the flatness of the solvent region typically found in macromolecular structures to the connectivity of regions of high electron density corresponding to the chains of polymers in these structures. Overall, the skewness of electron density stands out as the best of these measures (Table 3 ▶ and Fig. 2 ▶). Used in a simple Bayesian estimator, the correlation between map quality estimated with the skewness of electron density with true map quality is about 0.90, while the next-best estimator (the correlation of local r.m.s. density) gives a correlation of only 0.85. Combining the two yields the most useful estimator we have developed, with a correlation between estimated and actual map quality of 0.92 and an r.m.s. prediction error in map quality of 0.09.

With the exception of the mean figure of merit of phasing, which does not depend on the hand of the heavy-atom sub­structure, all the measures of map quality analyzed are remarkably good discriminators between maps calculated using the correct and inverse hands of the heavy-atom sub­structure (Fig. 3 ▶).

The *PHENIX AutoSol* wizard uses a combination of the skewness of electron density and the correlation of local r.m.s. density to form a Bayesian estimator of map quality. The *PHENIX AutoSol* wizard makes decisions about the heavy-atom substructures to pursue based on these map-quality estimates. Once density-modified maps are available, a model is built into the maps and the map–model correlation is used to identify the best overall solutions. This process yields density-modified electron-density maps of approximately the same overall quality as those obtainable with a perfect decision-making system (Fig. 5 ▶).

Our Bayesian estimates of map quality, while highly useful in evaluating experimental maps, are nevertheless not the best indicators of the quality of the corresponding density-modified maps. The map–model correlation obtained after preliminary model building is a better indicator of the quality of density-modified maps (Fig. 4 ▶ and Table 6 ▶).

In this work, we have ignored the resolution-dependence of the measures of map quality. This is made possible in part by the use of a high-resolution limit of 2.5 Å for all the calculations of map quality and is generally justified by the relatively small remaining resolution-dependence of most of the measures of map quality (Fig. 1 ▶). Nevertheless, it seems possible that some improvement in estimation of map quality might be obtained by including the resolution-dependence (or the effective overall isotropic displacement factor) of the data in the analysis. Additionally, we have assumed independence of the various measures of map quality in (8*a* [Disp-formula fd10]). We were not able to improve the estimates of map quality using a simple covariance-matrix approach to combining estimates of map quality, but other more sophisticated approaches, together with a much greater set of sample data, might lead to improved estimates of map quality.

## Figures and Tables

**Table 1:** Real-space measures of map quality tested in this work

			Expected properties	
Method	Symbol	Basis	Perfect map	Random map
Skewness of electron density	skew	High positive density and no negative density in a good map	Positive skewness	Near-zero skewness
Contrast of electron density	*c*	Solvent and macromolecule have different r.m.s. variation in densities	High contrast	Low contrast
Correlation of local r.m.s. density	*r*^2^_RMS_	Solvent region is contiguous so local r.m.s. is correlated with neighboring local r.m.s.	High correlation	Low correlation
Flatness of electron density	*F*	Solvent region has nearly flat electron density	High value of flatness	Low value of flatness
Number of regions enclosing high density	*N*_r_	Chains of a macromolecule can be represented by a few connected regions of density	Few (but extended) connected regions	Many short connected regions
Overlap of NCS-related density	*O*_NCS_	If NCS is present, NCS-related density is similar	High overlap	Low overlap

**Table 2:** Reciprocal-space measures of map quality tested in this work

			Expected properties	
Method	Symbol	Basis	Perfect map	Random map
Phase correlation from statistical density modification	*m*_DENMOD_	Phases from first cycle of density modification are unbiased and are correlated with experimental phases	High *m*_DENMOD_	Low *m*_DENMOD_
*R* factor from statistical density modification	*R*_DENMOD_	Amplitudes for a reflection can be calculated from phases and amplitudes of all other reflections and expected features of the map	Low *R*_DENMOD_	High *R*_DENMOD_
Density truncation	*r*^2^_TRUNCATION_	Much of the information in a map of a macromolecule consists of the density at points in the map near atomic positions	High *r*^*2*^_TRUNCATION_	Low *r*^*2*^_TRUNCATION_
Mean figure of merit of phasing	〈*m*〉	Estimates of accuracy of experimental phases are an approximate upper bound on quality of the map	High 〈*m*〉	Low 〈*m*〉

**Table 3:** Cross-validated prediction correlation

Quality measure(s)	Prediction correlation coefficient	R.m.s. prediction error
skew	0.90	0.10
*c*	0.78	0.15
*r*^2^_RMS_	0.85	0.12
*F*	0.80	0.14
*N*_r_	0.42	0.20
*m*_DENMOD_	0.80	0.10
*R*_DENMOD_	0.77	0.14
*r*^*2*^_TRUNCATION_	0.48	0.21
〈*m*〉	0.42	0.21
skew and *r*^2^_RMS_	0.92	0.09

**Table 4:** Correlation of prediction errors Values of *r* ^2^_MODEL_ were estimated for each measure of map quality using (7*a*) as in Fig. 3 ▶. The true values of *r* ^2^_MODEL_ were then subtracted, yielding prediction errors for each map for each measure of map quality. The correlation coefficients (*r* ^2^) of prediction errors among the various measures of map quality are listed.

	skew	*c*	*r*^2^_RMS_	*F*	*N*_r_	*m*_DENMOD_	*R*_DENMOD_	*r*^2^_TRUNCATION_	〈*m*〉
skew	1								
*c*	0.69	1							
*r*^2^_RMS_	0.60	0.82	1						
*F*	0.73	0.95	0.84	1					
*N*_r_	0.61	0.86	0.61	0.79	1				
*m*_DENMOD_	0.63	0.81	0.79	0.88	0.66	1			
*R*_DENMOD_	0.66	0.79	0.74	0.79	0.77	0.84	1		
*r*^2^_TRUNCATION_	0.54	0.82	0.63	0.71	0.88	0.61	0.76	1	
〈*m*〉	0.55	0.73	0.61	0.68	0.69	0.64	0.70	0.85	1

**Table 5:** Decision-making accuracacy for enantiomeric pairs The percentage of cases in which the higher (or lower, as appropriate) value of the quality measure is associated with the higher value of the actual map correlation coefficient with the corresponding model map. Only cases in which the actual map correlations differ by at least 0.05 are considered.

Quality measure(s)	Percentage of correct predictions
skew	0.98
*c*	0.94
*r*^2^_RMS_	0.95
*F*	0.94
*N*_r_	0.95
*O*_NCS_	0.90
*m*_DENMOD_	0.93
*R*_DENMOD_	0.94
*r*^2^_TRUNCATION_	0.97

**Table 6:** Decision-making accuracies in choosing the solution with the best experimental or density-modified map The percentage of correct predictions of best maps is the percentage of cases in which the solution with the highest value of the quality measure has a map correlation coefficient with the corresponding model map within 0.02 of that of the best obtained for any solution for that structure. The analysis is based on 372 sets of structure factors and associated maps obtained from 149 data sets as in Fig. 1 ▶, selecting the top-ranked 2–6 solutions and carrying out density modification with *RESOLVE* (Terwilliger, 2000 ▶) to yield density-modified maps. A model was built into each density-modified map using a rapid method for building helices and strands. If the value of the map–model correlation was less than 0.35, then the building procedure was repeated with a standard cycle of building using the methods in the *PHENIX AutoBuild* wizard (Terwilliger *et al.*, 2008 ▶) and the value of the map–model correlation from the full standard procedure was used. Only structures for which at least one model–map correlation was at least 0.20 are included in the analysis. The worst error in identification of the best maps is the largest value of the difference between the correlation coefficient of the best map with the corresponding model map and that of the map with the highest value of the quality measure.

	Percentage of correct predictions of best maps		Worst error in identification of best maps	
Quality measure	Experimental maps	Density-modified maps	Experimental maps	Density-modified maps
Bayesian estimate using skew and *r*^2^_RMS_ of experimental map	91	88	0.29	0.58
Map-model correlation for model built into density-modified map	87	92	0.40	0.26
